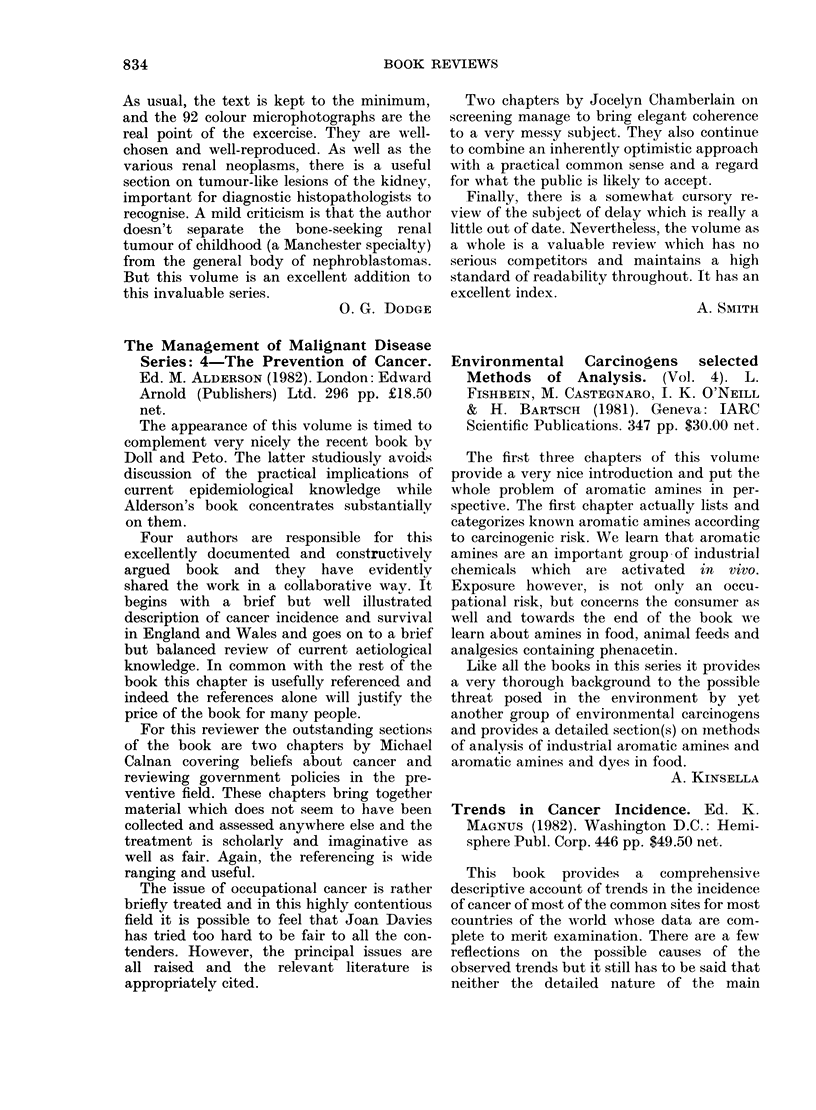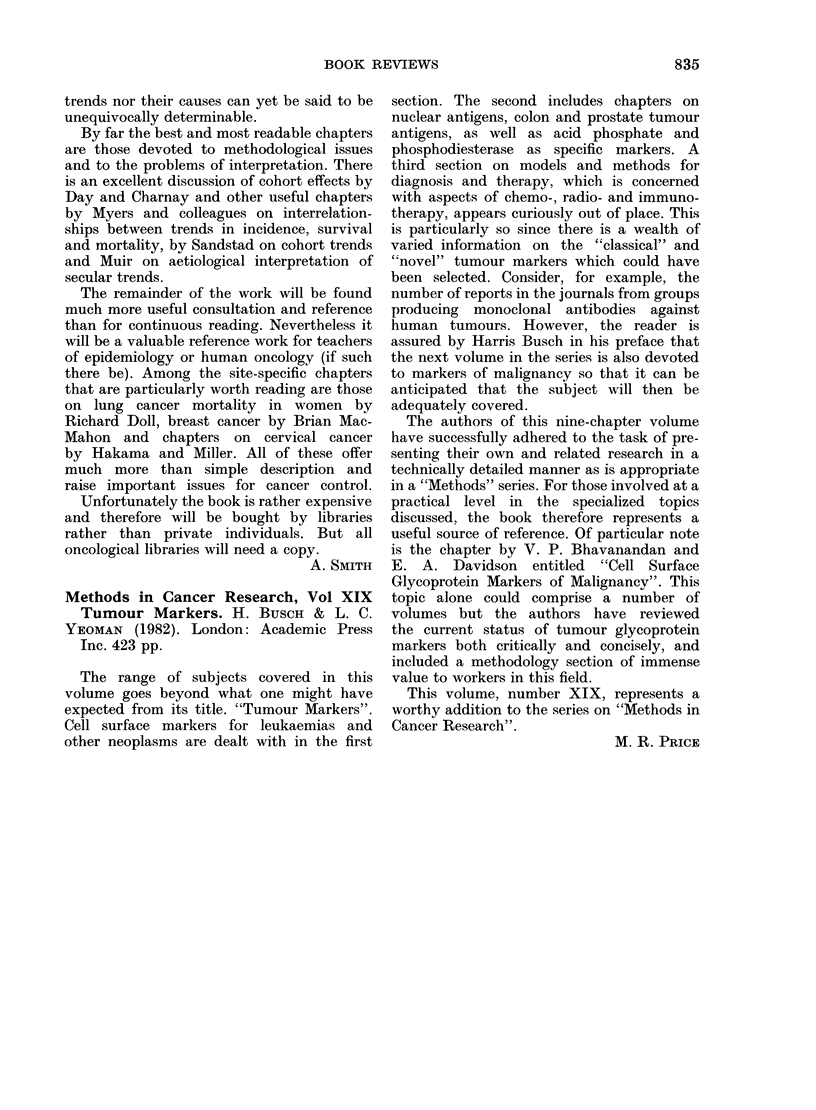# Trends in Cancer Incidence

**Published:** 1982-11

**Authors:** A. Smith


**Trends in Cancer Incidence.** Ed. K. Magnus (1982). Washington D.C.: Hemisphere Publ. Corp. 446 pp. $49.50 net.

This book provides a comprehensive descriptive account of trends in the incidence of cancer of most of the common sites for most countries of the world whose data are complete to merit examination. There are a few reflections on the possible causes of the observed trends but it still has to be said that neither the detailed nature of the main trends nor their causes can yet be said to be unequivocally determinable.

By far the best and most readable chapters are those devoted to methodological issues and to the problems of interpretation. There is an excellent discussion of cohort effects by Day and Charnay and other useful chapters by Myers and colleagues on interrelationships between trends in incidence, survival and mortality, by Sandstad on cohort trends and Muir on aetiological interpretation of secular trends.

The remainder of the work will be found much more useful consultation and reference than for continuous reading. Nevertheless it will be a valuable reference work for teachers of epidemiology or human oncology (if such there be). Among the site-specific chapters that are particularly worth reading are those on lung cancer mortality in women by Richard Doll, breast cancer by Brian MacMahon and chapters on cervical cancer by Hakama and Miller. All of these offer much more than simple description and raise important issues for cancer control.

Unfortunately the book is rather expensive and therefore will be bought by libraries rather than private individuals. But all oncological libraries will need a copy.

A. Smith